# Propolis can potentialise the anti-adhesion activity of proanthocyanidins on uropathogenic *Escherichia coli *in the prevention of recurrent urinary tract infections

**DOI:** 10.1186/1756-0500-4-522

**Published:** 2011-11-29

**Authors:** Jean-Philippe Lavigne, Xavier Vitrac, Louis Bernard, Franck Bruyère, Albert Sotto

**Affiliations:** 1 Institut National de la Santé et de la Recherche Médicale, U1047, Université Montpellier 1, UFR de Médecine, Chemin du Carreau de Lanes, Nîmes, France; 2 Department of Bacteriology, Caremeau University Hospital, Nîmes, France; 3 Polyphénols Biotech, University Bordeaux Segalen, ISVV, EA 3675, Villenave d'Ornon, France; 4 Department of Infectious Diseases, University Hospital, and University François Rabelais, Loire Valley, Tours, France; 5 Department of Urology, University Hospital, and University François Rabelais, Loire Valley, Tours, France

## Abstract

**Background:**

*Escherichia coli*, the main bacteria found in recurrent urinary tract infections (UTI), is now frequently resistant to several currently used antibiotic treatments making new solutions essential. In this study, we evaluated the association propolis and proanthocyanidins type A to reduce bacterial anti-adhesion activity of *E. coli *on urothelial cells.

**Results:**

This first double-blind, randomized, cross-over human trial included 5 volunteers that followed 6 different regimens with or without variable doses of cranberry and propolis with a washout period of at least 1 week between each regimen. Urine samples were collected at 0 h, 4-6 h, 12 h and 24 h after cranberry plus propolis or placebo capsule consumption. In vivo urinary bacterial anti-adhesion activity was assessed with a bioassay (a human T24 epithelial cell-line assay) and an in vivo *Caenorhabditis elegans *model. HPLC-PDA-MS was used to detect propolis and cranberry compounds in urine. Bioassays indicated significant bacterial anti-adhesion activity in urine collected from volunteers who had consumed cranberry plus propolis powder compared to placebo (*p *< 0.001). This inhibition was clearly dose-dependent, increasing with the amount of PACs and propolis equivalents consumed in each regimen. Results suggested that propolis had an additional effect with PACs and prevent a bacterial anti-adhesion effect over 1 day. An in vivo model showed that the *E. coli *strain presented a reduced ability to kill *C. elegans *after their growth in urine samples of patients who took cranberry plus propolis capsules. HPLC confirmed that propolis is excreted in urine.

**Conclusions:**

This study presents an alternative to prevent recurrent UTI. Administration of PACs plus propolis once daily offers some protection against bacterial adhesion, bacterial multiplication and virulence in the urinary tract, representing an interesting new strategy to prevent recurrent UTI.

## Background

Urinary tract infections (UTIs) are a widespread problem [1,2] whose recurrence is common in women, resulting in considerable morbidity, multiple antibiotic treatments and increased expenditures. Recently, uropathogenic *Escherichia coli *(UPEC), the major pathogen involved in these infections, acquired new resistance mechanisms against β-lactams and fluoroquinolones usually used to treat UTIs, leading to therapeutic deadlock of these frequent infections [3-10]. Therefore, new strategies to prevent or treat UTIs are essential.

Recent evidence suggested that ingestion of cranberries (*Vaccinium macrocarpon *Ait.) has been used for prevention of UTI [11-13]. A recent systematic review concluded that there is some positive clinical evidence that consumption of cranberry juice can reduce the number of symptomatic UTIs in women over a 12-month period [12]. Research showed that consuming cranberry products may prevent adhesion of *E. coli *strains to the uroepithelium [14-16], notably multidrug resistant strains [17], thus interfering with this important initial step in the infection process [18]. The proanthocyanidins (PACs) in cranberry, in particular the A-type linkages, have been implicated as important inhibitors of primarily P-fimbriated *E. coli *adhesion to uroepithelial cells in vitro [13,19-21] and ex vivo [22,23].

Only cranberry juice with A-type PACs prevented bacterial adhesion, and molecular weight of PACs could potentially impact the bacterial anti-adhesion activity. Indeed, Foo et al. [20] showed that the active compounds consisted predominantly of epicatechin units with mainly degree of polymerization of 4 and 5 containing at least one A-type linkage. Thus, the active components in cranberry should be type-A oligomeric procyanidins.

Propolis is a resinous material collected by bees from exudates and buds of plants, then mixed with wax and bee enzymes. Propolis's antimicrobial activities are well documented against different bacteria [24]. In vitro propolis may act directly on microorganisms, and in vivo it may stimulate the immune system, activating the killing of bacteria. Propolis may show additional effects with antimicrobial drugs (fluoroquinolones and β-lactams).

The objective of this study was to evaluate the association of proanthocyanidins and propolis concerning bacterial anti-adhesion activity, to evaluate additional dosage regimes and collection time-periods following ingestion of the propolis and PACs-standardized cranberry powder.

## Results

### Effects detected by ex vivo assays

For PACs regimen, ex vivo epithelial cell adhesion assay indicated highly significant reductions in bacterial adhesion to T24 cells compared to placebo (*p *< 0.001) following the consumption of cranberry dosages containing 60 mg of PACs (Table 1). An adhesion index (AI) corresponding to the mean number of adherent bacteria per cell for 100 cells was calculated. The AI of bacteria grown in urine samples collected after consumption of cranberries with 60 mg PACs was significantly lower than AI following the dose with placebo (*p *< 0.001). The analysis also confirmed a time dependent-effect, with maximum effect at 6 h compared to 24 h with 60 mg of PACs (*p *< 0.001) (Table 1).

**Table 1:** Urinary bacterial adhesion to T24 cells expressed as Adhesion Index (AI) following consumption of increasing doses of cranberry powder of proanthocyanidins (PACs) with or without propolis vs. placebo. The results are representative of at least three independent trials

AI Median [Range]									
PAC* (mg)	-	36	60	60	60	72	90	120	144
**Propolis (mg)**	**-**	**-**	**-**	**170**	**192**	**-**	**255**	**340**	**-**

4-6 h	21.5 [15-28]	10.5 [5-16]	4.7 [0-13]	3.0 [0-8]	3.5 [0-10]	5.9 [1-11]	2.1 [0-7]	1.3 [0-6]	2.5 [1-6]

12 h	22.5 [16-30]	14.4 [8-19]	12.6 [5-18]	13.7 [5-25]	7.5 [0-14]	5.9 [1-9]	2.9 [15-5]	1.8 [0-6]	ND

24 h	22.3 [15-29]	16.0 [10-22]	21.5 [15-26]	17.1 [12-29]	18.5 [10-25]	14.5 [2-25]	17.4 [10-25]	3.5 [0-10]	9.9 [2-15]

Reference	This study	[17, 23]	This study	This study	This study	[17,23]	This study	This study	[23]

*Expressed as vanillin-H_2_SO_4 _equivalents

Range of values are indicated into [square brackets]

ND not determined

For PACs plus propolis regimens, there was a dose-dependent decrease in bacterial adhesion with cranberry and propolis intake. The AI of bacteria grown in urine samples collected after consumption of cranberry with 60 mg PACs and 192 mg propolis was significantly lower than the AI following the dose with 60 mg PACs and 170 mg propolis in urine collected at 12 h (*p *< 0.001). Moreover, there was a time-dependent effect. AI of bacteria in urine collected at 6 h and 24 h was significantly different following all regimens (*p *< 0.001), except for 120 mg PACs and 340 mg propolis. For this last regimen, the AI at 24 h remained significantly low (AI median < 4) compared to the other regimens (*p *< 0.001).

### Reduction of *E. Coli *virulence

The mean survival time (LT50) for worms was significantly increased to 5-6 days with *E. coli *grown in urine samples collected after cranberry or cranberry plus propolis intake compared to LT50 for strains grown in urine samples collected after placebo intake (3-4 days) (*p *< 0.001) (Table 2). In urine collected after 6 h, no difference was detected between the virulence of *E. coli *strain grown in urine collected after consumption of PAC and propolis. However, there was a significant decrease of LT50 with *E. coli *grown in urine samples collected after the intake of 120 mg PACs and 340 mg of propolis after 24 h compared to all other regimens at the same time (*p *< 0.001).

**Table 2:** In vivo kinetics of killing of *C.elegans *infected by *E. coli *grown in urine samples after consumption of the different proanthocyanidins plus propolis regimens.

Strains	PACs mg	Propolis mg	Collection time	LT50	LT100	p		
						
						T0 vs. others	test 1 vs. test 2	test 1 vs. test 4
NECS978323	-	-	0	4.2 ± 0.2	7.4 ± 0.6	NA		

test 1	60	-	4-6 h	6.5 ± 0.1	11.5 ± 0.5	< 0.001	NS	
	
	60	-	12 h	5.6 ± 0.2	9.5 ± 0.5	< 0.001	NS	
	
	60	-	24 h	4.2 ± 0.2	7.3 ± 0.7	NS	NS	

test 2	60	170	4-6 h	6.0 ± 0.1	11.3 ± 0.7	< 0.001	NS	
	
	60	170	12 h	5.5 ± 0.2	9.3 ± 0.7	< 0.001	NS	
	
	60	170	24 h	5.0 ± 0.2	7.8 ± 0.8	NS	NS	

test 3	60	192	4-6 h	6.1 ± 0.1	11.2 ± 0.8	< 0.001		
	
	60	192	12 h	5.5 ± 0.2	9.5 ± 0.5	< 0.001		
	
	60	192	24 h	5.3 ± 0.2	8.5 ± 0.5	0.02		

test 4	90	255	4-6 h	6.3 ± 0.3	11.3 ± 0.7	< 0.001		NS
	
	90	255	12 h	5.3 ± 0.3	9.5 ± 0.5	< 0.001		NS
	
	90	255	24 h	5.8 ± 0.2	9.0 ± 1.0	< 0.001		< 0.001

test 5	120	340	4-6 h	6.0 ± 0.1	11.8 ± 0.8	< 0.001		
	
	120	340	12 h	6.2 ± 0.2	11.8 ± 0.8	< 0.001		
	
	120	340	24 h	6.3 ± 0.3	11.5 ± 0.5	< 0.001		

test 6	-	-	4-6 h	4.1 ± 0.2	7.2 ± 0.8	NS		
	
	-	-	12 h	3.9 ± 0.1	7.0 ± 0.5	NS		
	
	-	-	24 h	4.2 ± 0.3	7.3 ± 0.7	NS		

OP50	-	-	-	7.5 ± 0.5	13.3 ± 0.7	< 0.001		

The results are representative of at least three independent assays. OP50 is an avirulent *E. coli *strain used as control of the vitality of worms. *E. coli *NECS978323 was the strain used in the study. It was used as control (without treatment)

NS not significant

The number of *E. coli *CFU within the nematode gut varied around 10^5^-10^6 ^bacteria per worm for each strain 72 h after ingestion without significant difference (Table 3) confirming ingestion and proliferation of *E. coli *isolates in the *C. elegans *intestine.

**Table 3:** Evaluation of the number of bacteria within the *C.elegans *digestive tract.

Strains	PACs mg	Propolis mg	Collection time	Median CFU [range]/nematodes after 72 h
NECS978323	-	-	0	8.1 × 10^5 ^[7.0-9.3 10^6^]

test 1	60	-	4-6 h	3.3 × 10^5 ^[2.5-4.0 10^6^]

	60	-	12 h	3.5 × 10^5 ^[2.1-5.0 10^6^]

	60	-	24 h	5.2 × 10^5 ^[4.4-6.1 10^6^]

test 2	60	170	4-6 h	4.4 × 10^5 ^[4.0-4.8 10^6^]

	60	170	12 h	5.1 × 10^5 ^[4.8-5.4 10^6^]

	60	170	24 h	6.0 × 10^5 ^[4.9-7.2 10^6^]

test 3	60	192	4-6 h	2.3 × 10^5 ^[2.0-3.8 10^6^]

	60	192	12 h	4.8 × 10^5 ^[4.2-5.4 10^6^]

	60	192	24 h	3.0 × 10^5 ^[2.5-3.5 10^6^]

test 4	90	255	4-6 h	2.7 × 10^5 ^[1.0-4.2 10^6^]

	90	255	12 h	4.3 × 10^5 ^[3.5-5.1 10^6^]

	90	255	24 h	3.7 × 10^5 ^[3.2-4.0 10^6^]

test 5	120	340	4-6 h	2.5 × 10^5 ^[1.9-3.0 10^6^]

	120	340	12 h	3.8 × 10^5 ^[2.2-5.4 10^6^]

	120	340	24 h	1.8 × 10^5 ^[1.0-3.1 10^6^]

test 6	-	-	4-6 h	6.0 × 10^5 ^[4.9-7.2 10^6^]

	-	-	12 h	4.5 × 10^5 ^[3.9-5.1 10^6^]

	-	-	24 h	7.1 × 10^5 ^[6.8-7.5 10^6^]

OP50	-	-	-	6.8 × 10^5 ^[6.0-7.5 10^6^]

After 72 h of infection, the *C. elegans *were washed and ground, and dilutions of the resulting suspension were plated on selective media. The number of CFU per worm of the different strains alone or in combination was calculated. Three replicates were performed for each bacterial combination. *E. coli *NECS978323 was the strain used in the study. It was used as control (without treatment). No statistical difference could be demonstrated

Range of values are indicated into [square brackets]

### Identification and determination of native compounds and metabolites in capsules and urine

In the capsules, the content of A-type proanthocyanidins oligomers (dimers and trimers) expressed as procyanidin A2 equivalents were 1 mg per capsule. In urine, no A-type proanthocyanidin (neither in native form or metabolised) was detected.

Concerning the potential active compounds from propolis, we focused on galangin and pinocembrin, the two main flavonoids found in propolis extracts from various origins [25]. Their content in capsules was found to be 1.41 mg per capsule. Free forms of galangin and pinocembrin were present in very low amounts in urine indicating that these compounds are excreted mainly in a conjugated form. The time course of the concentration of the free forms are shown in Table 4. The concentration of the conjugates of pinocembrin in urine was relatively constant during the first 12 h, and reached their highest level 24 h after administration. In contrast, the conjugates of galangin in urine were maximal 4-6 h after administration, and appeared to be maintained during the next collecting times. In addition, pinocembrin seems to be excreted more efficiently than galangin since the ratio galangin/pinocembrin was higher in capsules than in urine (data not shown).

**Table 4:** Time courses of contents of pinocembrin and galangin in β-glucuronidase/sulfatase treated urine collected after intake of capsules containing cranberry and propolis

	4-6 h	12 h	24 h
Pinocembrin (mg/L)	0.83	0.71	1.85

Galangin (mg/L)	0.19	0.09	0.14

## Discussion

The increasing resistance of *E. coli*, bacteria most frequently isolated during UTI to diverse antibiotics is an international public health problem [7-10]. The very common recurrence of UTI in women requires new antibiotic treatments, promotes the emergence of new mechanisms of resistance, inducing a vicious circle. Finding new ways to prevent and/or treat these infections is essential. A number of strategies minimizing antibiotic exposure have been used attempting to prevent recurrent UTI. This study shows the effect of propolis and cranberry (*Vaccinium macrocarpon*) in ex vivo and in vivo anti-adhesion assays.

When analysing different results obtained after cranberry intake by our team [17,23], we established that the minimal PACs dose with an observed anti-adhesion effect is 36 mg. Moreover, we could see an anti-adherent effect dose and time-dependent inversely proportional to the PACs dose. This study shows that the dose-dependent and time-dependent effects are also observed when propolis was added to PACs (Table 1).

Moreover, an additional effect of propolis with PACs was clearly noted. At 60 mg PACs and 170 mg of propolis, no supplementary effect was observed on AI compared to the consumption of 60 mg PACs alone. However, with 192 mg of propolis, a significant anti-adhesion effect is observed at 12 h (*p *< 0.001) for the same PACs content. With 120 mg of PACs and 340 mg of propolis a nycthemeral effect was clearly noted: the AI observed at 24 h is significantly higher than the AI detected for 144 mg PACs alone (*p *< 0.001). An additional anti-adherence action of PACs plus propolis is observed, especially since the amount of absorbed PACs is important.

We could speculate that in addition to the anti-adhesion activity of PACs, propolis killed the bacteria, decreasing the number of adherent bacteria and facilitating the activity of PACs. The PACs contained in cranberry inhibit *E. coli *adhesins notably against P-fimbriae [15,20]. Adhesion is the first step of bacterial virulence, ensuring survival and establishing replication. To demonstrate the effect of PACs on bacterial virulence, we used an in vivo killing nematode model validated by different teams [17,26,27]. This nematode lives in soil and eats environmental bacteria. It is a simple and easy to handle model that can be used to evaluate the virulence of bacterial pathogens. It is increasingly being used to study host-pathogen interactions and has helped identify basic evolutionarily conserved pathways associated with microbial pathogenesis. In particular, this has revealed important factors of the host response with remarkable parallels in higher organisms. This organism's short (2-3-weeks) life span facilitates host-bacteria interaction analysis, offering an ideal compromise between complexity and tractability. We determined that the reduced ability of *E. coli *strain to kill worms correlates with the consumption of cranberry plus propolis capsules. Bacteria grown in the urine of individuals consuming cranberry capsules was not able to adhere to worms, thus leading to a reduced killing of the worms despite the presence and proliferation of *E. coli *strain in the *C. elegans *intestine. *E. coli *were localized within the nematode gut, but could not exert any virulent effect due to an inability to establish infection and loss of virulence. In our in vivo experiment we again demonstrated the additional action of PACs and propolis: a significant decrease of LT50 with *E. coli *grown in urine samples collected after the intake of 120 mg PACs and 340 mg of propolis observed after 24 h compared to all other regimens over the same time, even for 144 mg PACs.

In our study we evaluated the presence of the main propolis flavonoids (galangin and pinocembrin) and PACs in capsules and urine samples by HPLC-PDA-MS. Currently there is no universally accepted standard method for quantification of cranberry PACs in products [28]. Quantification of PACs using colorimetric methods (DMAC reagent, European Phamacopeae, Bate-Smith, vanillin reagent) can lead to erroneous and unreproducible results, absolutely not specific to the type of linkage of proanthocyanidins units (A-or B-type). Since many of these methods were used to determine the total PACs content in the capsules used in the published clinical studies, little is known about the specific A-type PACs content. In the present study, we analysed the metabolites of the administrated cranberry and propolis in urine before and after treatment with β-glucuronidase/sulfatase. No PACs were detected. This result, in agreement with literature [29], lacks evidence that PACs may be biotransformed to unique biologically active urinary metabolites, or even reach the urine intact as dimers or trimers. Indeed, little is known about the metabolic route of A-type PACs in humans, especially if they are degraded in the gut, and/or not assimilated in any quantity, and do not reach the urinary tract intact. Because the putative active proanthocyanidins are possibly not absorbed and/or may be metabolized before reaching the urinary tract, it is probable that other cranberry compounds or their metabolites are active. Conversely, we demonstrated the presence of metabolites of propolis in urine samples collected at various times after administration of capsules containing cranberry and propolis. Pinocembrin and galangin were present in urine, which suggests that these two compounds are absorbed in the blood and excreted as glucuronide and/or sulfo-conjugates. Their high concentrations in urine collected 24 h after capsule administration could be responsible for the observed effects. The antibacterial activities of these two metabolites are part of our ongoing research. A recent study showed that galangin isolated from *Alpinia officinarum *Hance have the potential to reverse bacterial resistance to β-lactam antibiotics against penicillin-resistant *Staphylococcus aureus *[30].

The main limitation of this study is the use of ex vivo experiments to observe the effect. Further human trials are needed to correlate the level of ex vivo anti-adhesion activity with the prevention of clinical UTI.

## Conclusions

Administration of PACs-standardized cranberry plus propolis powder could offer a protection against bacterial adhesion in the urinary tract and could represent an additional means to help prevent infections.

## Methods

### Chemicals

Procyanidin A2 was purchased from Extrasynthèse (France). β-glucuronidase/sulfatase, galangin and pinocembrin were purchased from Sigma-Aldrich (France). Other chemicals were of HPLC grade.

The propolis extract used in this study is an hydroalcoholic extract of blended propolis from various origins (China, Europa) mixed with carob in a proportion (60/40, w/w). This extract was standardized to contain 2% galangin.

The cranberry extract was obtained by purification of the polyphenolic fraction of a cranberry juice, and was standardized to contain 20% A-type proanthocyanidins.

### Bacterial strains

An uropathogenic *E. coli *strain previously isolated from a patient with UTI (NECS978323) [17] with P-fimbriae *papG *and type-1 pili was utilized. To allow the direct observation of adherent bacteria by fluorescence microscopy, NECS978323 was genetically modified to express green fluorescent protein [31].

### Healthy volunteers

Even if cranberry is a supplement food, the study has been approved by our local research ethic committee (South Mediterranean III) and has been conducted according to the principles expressed in the Declaration of Helsinki. Five healthy volunteers with normal diets (25-48 years of age) were included in this double-blind, randomised, placebo-controlled and cross-over study. Exclusion criteria included antibiotic use within 6 months prior to the study, pregnancy, known allergy or intolerance to cranberry/propolis products, routine consumption of any food supplements consisting of vitamins, minerals or trace elements. All volunteers signed informed consent. Throughout the study, volunteers were instructed not to alter their dietary or lifestyle habits. However, during the capsule consumption, volunteers were told to avoid all *Vaccinium*-containing foods, drinks and supplements.

### Ex vivo and in vivo protocol

The study used cranberry powder capsules, cranberry and propolis powder capsules and placebo capsules composed of colloidal silica, magnesium stearate, cellulose and gelatin. The capsule dosages were standardized, delivering 15 mg of PACs equivalents (vanillin-H_2_SO_4 _method) in cranberry powder alone or associated with 42.5 mg of propolis and/or 48 mg of propolis. A propolis-only capsule was not tested, as propolis alone has no anti-adhesion activity [24]. To avoid confusion, all PACs dosages reported in this paper were expressed as vanillin-H_2_SO_4 _equivalents. Data from previous studies cited in this article [17,23] were obtained using the Old-DMAC method. In order to make the results more readable, these data were converted to vanillin-H_2_SO_4 _equivalents, while multiplying the Old-DMAC values by a factor of 2, which is generally well-accepted.

Each volunteer received successively six regimens (always 8 capsules) distributed in random order, consisting of: (1) 4 capsules of cranberry and 4 capsules of placebo; (2) 8 capsules of placebo; (3) 4 capsules of cranberry plus propolis (at 42.5 mg) and 4 capsules of placebo; (4) 4 capsules of cranberry plus propolis (at 48 mg) and 4 capsules of placebo; (5) 6 capsules of cranberry plus propolis (at 42.5 mg) and 2 capsules of placebo; (6) 8 capsules of cranberry plus propolis (at 42.5 mg); with a washout period of at least 1 week between each regimen. Volunteers consumed the capsules in the morning at 8:00. The first urine samples were collected between 12:00-14:00 following capsule consumption and then pooled. The second urine samples were collected at 20:00. The third collections were made the following morning (8:00). Control urine samples were collected at the beginning of the study, prior to consuming the capsules. Remaining samples were centrifuged at 4000 g for 15 min., sterilized by filtration (0.45 μm), and stored at-20°C.

### Bacterial anti-adhesion assays

In the ex vivo urine assays, bacterial adhesion was evaluated using the human T24 epithelial cell-line (ATCC HTB-4). The technology was developed using fluorescent NECS978323 to enhance detection of strain adhesion as previously described [23]. An adhesion index (AI) corresponding to the mean number of adherent bacteria per cell for 100 cells was calculated. This index was expressed as the mean of at least three independent assays.

### Nematode killing assay

The *Caenorhabditis elegans *test is based upon the capacity of pathogens ingested by nematodes to infect and kill [17]. The survival nematodes fed on *E. coli *strain is an indirect marker of their potential virulence. This assay was carried out as described by Lavigne et al. [17] using Fer-15 mutant line. At least three replicates repeated 5 times were performed for NECS978323 strain. Lethal Time 50% (LT50) and death (LT100) corresponded to time required to kill 50% and 100% of the nematode population, respectively. The number of bacteria within the *C. elegans *digestive tract was carried out as described [17].

### HPLC-PDA-MS analysis of propolis and cranberry phenolic compounds in capsules and urine

The main propolis flavonoids (galangin and pinocembrin) and cranberry PACs (A-type procyanidins) in capsules and urine samples were analysed by HPLC-PDA-MS (Agilent Technologies). For capsules analysis, 1 mg of powder was dissolved in 1 ml of 50% aqueous MeOH and the solution was filtered (0.45 μm) before HPLC analysis. For the urine sample analysis, 2 ml of urine was extracted by shaking for one hour with 5 ml of ethyl acetate (2 times). The two ethyl acetate solutions were combined, evaporated to dryness and the residue re-suspended in 200 μl of 0.1% formic acid in 50% aqueous MeOH before HPLC analysis. Conjugates were identified on the basis of enzymatic hydrolysis with glucuronidase (5000 units of β-glucuronidase and 40 units of sulfatase). HPLC separation of samples was performed using a Prontosil C18 reverse phase column (250 mm×4 mm, 5 μm particle size). Chromatographic data were acquired with a photo-diode array detector set at 260 and 280 nm, coupled with an Esquire 3000 plus MS (Bruker Daltonics, Germany) equipped with an electro-spray ionization (ESI) source. Identification was based on the MS and MS/MS spectra collected over the chromatographic peaks and their comparison with spectra of standards (pinocembrin, galangin, procyanidin A2).

### Statistical analysis

The quantitative variables were described by median values, range and mean, as well as standard deviation. Comparisons between different regimens were evaluated using a two-way ANOVA procedure. The normality of the distribution was checked in each class by the Shapiro-Wilks test. A Student-Newman-Keuls Test evaluated the statistical difference between each pair of groups. The entire survival curves in nematode killing assays were compared using a Cox regression comparing the timing of mortality between treatments. A value of *p *≤ 0.05 given by the SAS^®^/ETS software (version 8.1; SAS Institute Inc, Cary, NC, USA) was considered statistically significant.

## Competing interests

JPL, FB, LB, AS are consultant for Nutrivercell. JPL and AS have participated in a trial organized by Pharmatoka.

Nutrivercell and Pharmatoka had no role in the study design, data collection and analysis, decision to publish or preparation of the manuscript.

## Authors' contributions

JPL and AS conceived of the study, participated in its design and coordination, carried out the ex vivo and in vivo assays and drafted the manuscript. LB and FB participated in the design of the study and coordination and helped to draft the manuscript. XV participated in the design of the study and its coordination, carried out the HPLC-PDA-MS assays and helped to draft the manuscript. All authors read and approved the final manuscript.
